# Molecular heterogeneity of glucose-6-phosphate dehydrogenase deficiency in neonates in Wuhan: Description of four novel variants

**DOI:** 10.3389/fgene.2022.994015

**Published:** 2022-09-21

**Authors:** Shanshan Shen, Qian Xiong, Wenqian Cai, Rui Hu, Bin Zhou, Xijiang Hu

**Affiliations:** Wuhan Children’s Hospital (Wuhan Maternal and Child Healthcare Hospital), Tongji Medical College, Huazhong University of Science and Technology, Wuhan, Hubei, China

**Keywords:** G6PD variant, G6PD genetic, newborn screening, novel variants, Wuhan

## Abstract

Glucose-6-phosphate dehydrogenase (G6PD) deficiency is one of the most common X-linked enzymopathies caused by *G6PD* gene variant. The aim of this study was to investigate the molecular epidemiological characteristic of the G6PD deficiency among newborn screening population in Wuhan region. A total of 430,806 healthy neonates in Wuhan area of China were screened for G6PD deficiency from November 2016 to December 2021. The positive samples were further detected with gene analysis. Among the 957 neonates with abnormal G6PD enzyme activity, the prevalence of G6PD deficiency in Wuhan was calculated as 0.22%. 38 genotypes were found and the top 5 frequencies of *G6PD* gene variants were c.1388G > A, c.1376G > T, c.95A > G, c.1024C > T and c.871G > A. Seven rare single variants (c.25C > T, c.152C > T, c.406C > T, c.497G > A, c.679C > T, c.854G > A and c.1057C > T) and two rare multiple variants (IVS-5 637/638T del/c.1311C > T/1365-13T > C and c.406C > T/c.1311C > T/1365-13T > C) were discovered in this study. In addition, four novel variants (c.49C > T, c.691G > A, c.857A > T and c.982G > A) were detected out in our cohort, which have never been reported before. The result indicated that a rich diversity of G6PD genetic variants in Wuhan region, also had its own regional characteristic. Our data provided the basic knowledge for future prevention and research of G6PD deficiency and the findings will be useful for genetic counseling and prenatal diagnosis of G6PD deficiency in the Wuhan region.

## Introduction

Glucose-6-phosphate dehydrogenase (G6PD) deficiency, the most common red blood cell enzyme deficiency affecting more than 400 million people worldwide, may cause a large spectrum of diseases, including favism, hemolytic anemia, chronic nonspherocytic hemolytic anemia (CNSHA), and neonatal hyperbilirubinemia, which is closely linked to neonatal kernicterus that can lead to death (Luzzatto et al., 2020; Wang et al., 2021). G6PD deficiency is not a disease but an inherited condition where only gene therapy can rectify this. However, unless the individual having the deficiency is with CNSHA, it is pretty much asymptomatic until he/she is exposed to external oxidants such as anti-malarial primaquine or sulfa-based drug (Frank, 2005; Cappellini and Fiorelli, 2008).

G6PD is encoded by the *G6PD* gene located in the Xq28 region, with a full-length sequence of 18.5 Kb, consisting of 13 exons and 12 introns, encoding 515 amino acids. Variants in the *G6PD* gene affect the stability and activity of the enzyme causing irreversible oxidative damage in the red blood cells (RBCs) with a wide range of biochemical and clinical phenotypes (Howes et al., 2013). To date, more than 400 *G6PD* biochemical variants and 217 gene mutations have been identified worldwide (Mason et al., 2007; Minucci et al., 2012; Gómez-Manzo et al., 2016; Luzzatto et al., 2020). Most *G6PD* variants were reported with point mutation, which could induce different levels of enzyme activity, and approximately 35 different molecular abnormalities have been identified in the Chinese population (Liu et al., 2020). Previous studies have revealed that *G6PD* genotype varied widely in different regions and ethnic groups, with higher prevalence in the southern regions of the Yangtze River Valley (Yang et al., 2015), particularly in Guangdong, Guangxi, Yunnan, Hainan, Guizhou, and Sichuan provinces. Despite previous reports about the population prevalence and variant spectrum of G6PD in most regions of south China (Yang et al., 2015; Chen et al., 2018; He et al., 2018; Lin et al., 2018; Zhang et al., 2018; Liu et al., 2020), there were only a few scattered reports of G6PD variants in north China.

Wuhan, covering an area of 8569.15 km^2^, is a major city in central China and located in Hubei Province in the middle reaches of the Yangtze River. As of the 7th Nationwide Census in 2020, the resident population in this city reached 12.3265 million, including over 1 million college students, suggesting the entry of many new comers from all over the country, with a complex genetic background and a rapid floating characteristic, leading to the occurrence of many cases of G6PD deficiency in Wuhan, such as a novel heterozygous variant c.141G > C in a girl with favism as reported in our previous study (Shen et al., 2020). In China, routine assays for newborn disease screening started relatively late, with G6PD deficiency being first included in newborn screening panels in 2008. At the end of 2016, the newborn screening program of G6PD deficiency was carried out comprehensively in the whole region of Wuhan, and the prevalence of G6PD deficiency in Wuhan neonates was found to be 0.18% in our previous survey (Shen et al., 2019). However, little information is available about the molecular characterization of G6PD deficiency in this region. Therefore, the aim of this study was to explore the genotype and distribution characteristics of G6PD deficiency in the Wuhan neonatal cohort born from 2016 to 2021 and analyze the potential effects of the novel variants found in our cohort using the in-silico tools. The results of this study are an important supplement to the nation-wide molecular epidemiological survey of G6PD deficiency in central China.

## Materials and methods

### Samples

A total of 430,806 healthy neonates (male: 230,152; female: 200,654) in Wuhan were selected for the G6PD deficiency screening program and they were recruited by Wuhan Neonatal Diseases Screening Center from November 2016 to December 2021. Healthy, non-distressed neonates, ≥35 weeks of gestation were enrolled. Infants with significant distress, congenital anomalies, or who had received blood transfusion were excluded. Informed consent was obtained from the subjects’ parents before collecting blood samples. This study was approved by the Medical Ethics Committee of the Human Subjects of Wuhan Children’s Hospital (Wuhan Maternal and Child Healthcare Hospital).

### Glucose-6-phosphate dehydrogenase deficiency screening

The preliminary screening was carried out from November 2016 to December 2021. On the third day post birth, blood specimens were collected from the heels of neonates according to the “Technical Specifications for Neonatal Disease Screening (2010 edition)”, and then were dripped on No. 903 filter paper. The blood spots with the diameter of >8 mm were stored at 4–8°C and timely delivered to the Neonatal Disease Screening Department of Wuhan Children’s Hospital after complete infiltration and drying at room temperature, followed by collecting a blood spot with a diameter of 3 mm for detection. A modified Fluorescent Spot Test (FST) was used to assess G6PD activity using the neonatal G6PD mensuration reagent kit (Fenghua China Guangzhou Co., Ltd.). This commercially available procedure is a spectrophotometric method determining G6PD activity by measuring the NADPH molecule formation. The fluorescence signal was measured with a Auto TRFIA-4 semi-auto time-resolved fluoroimmunoassay analyzer (Fenghua China Guangzhou Co., Ltd.). The enzyme activity was expressed as U/g Hb, with the cutoff value ≥2.5 U/g Hb for normal G6PD enzyme activity. The G6PD deficiency positive cases preliminarily screened were immediately recalled to collect the blood samples.

### DNA analysis and G6PD/6PGD ratio testing

The neonates suspected with G6PD deficiency were recalled for tested by both *G6PD* mutations analysis and G6PD enzymatic activity. The improved G6PD Nitroblue tetrazolium (NBT) Quantification Ratio Kit (Fenghua China Guangzhou Co., Ltd) was used to the quantitative evaluation of G6PD activity according to the manufacturer’s instructions. G6PD/6PGD ratio of ≤1.0 were classified as G6PD deficient. For the detection of *G6PD* mutations, genomic DNA was collected from peripheral blood leukocytes using a blood genomic DNA extraction kit (Tiangen Bio-Tech Co. Ltd., Beijing, China) as instructed by the manufacturer. The Sanger method was used for DNA sequencing of the 12 coding exons and intronic flanking regions of the G6PD gene (NCBI: G6PD NG_009015, NM_001042351). Table 1 shows the detailed information of primers (exon 1 does not encode protein). PCR amplification was carried out in a 25 μL reaction system containing 20 μL of PCR mix and 5 μL (5 ng/μL) DNA template under the conditions of 95°C for 5 min, followed by 32 cycles of 94°C for 30 s, 60°C for 30 s and 72°C for 30 s, and a final extension of 7 min at 72°C. Subsequently, the PCR amplification products were purified and analyzed by direct sequencing using an ABI 3130 Sequencer (Perkin ElmerApplied Biosystems, Wellesley, MA, United States), followed by analyzing the sequencing results using the Chromas and GeneScan Analysis Software version 3.7. Each sample was sequenced on both sense and antisense strands.

**TABLE 1:** The detailed information of primers.

Exon	Primer sequences	Tm	Product length, bp
2	F: GCC​GTT​CAC​AAG​GAG​TGA​TT	60	418
	R: CAG​GTA​GAG​CCG​GGA​TGA​T		
3 + 4	F:GCTTGTGGCCCAGTAGTGAT	60	665
	R: ACA​GAG​GCC​AGA​TTT​CAG​GA		
5	F: CTG​CTA​AGA​TGG​GGC​TGA​AC	60	554
	R: GAAAGGCGGTGTTTCGTG		
6	F: GTG​TTG​AGC​CAG​AGG​GTC​AT	60	488
	R AGG​TGG​AGG​AAC​TGA​CCT​TG		
7	F: TGG​TAC​TCA​GGA​GCC​TCA​CC	60	400
	R:CTGATAGCTCAGACACTTAGGTTTT		
8	F: GCA​TCA​CCA​TGT​CCT​TCC​TT	60	438
	R: CAA​CTT​GGG​CTT​CAT​GAC​TG		
9	F: GCC​TCA​GCT​TGT​TCA​TCA​GA	60	555
	R:AGGATGAAGGGCACCCCTA		
10	F: AGG​TGG​GAT​GGT​AGG​TGA​TG	60	497
	R: TTC​ACG​TTC​TGT​GAG​GGA​GA		
11 + 12	F: CCT​GAC​CTA​CGG​CAA​CAG​AT	60	571
	R: CCA​CTT​GTA​GGT​GCC​CTC​AT		
12 + 13	F: CCT​CAT​CCT​GGA​CGT​CTT​CT	60	599
	R: TAG​CTG​GGC​TCG​GGT​AGT​AG		

### *In silico* analysis

For the novel variants identified in Wuhan birth cohort with G6PD deficiency, the conservation of peptide sequences around the affected residues was assessed by aligning different species and human G6PD protein sequences with ClustalW2. The potential effect of the variant on the enzyme structure was analyzed using the bioinformatic tools of Sorting Intolerant from Tolerant (SIFT), Polymorphism Phenotyping v2 (PolyPhen-2), and Mutation Taster. Structural analysis was performed using the X-ray structure of G6PD deposited in the Protein Data Bank (PDB IDs: 2BHL and 2BH9), and the protein models of novel variants were created with SWISS-MODEL and visualized using Swiss PDB Viewer.

### Statistical analysis

All data were analyzed by SPSS 18.0. The incidence was expressed as a percentage (%) and the comparison between groups were done using χ^2^ testing, *p* <0.05 was considered to be statistically significant.

## Results

### Preliminary screening results

From November 2016 to December 2021, a total of 958 cases were preliminarily screened as positive from the 430,806 healthy neonates, and the prevalence of G6PD deficiency in Wuhan was calculated as 0.22%, including 863 male cases (0.37%, 863/230152) and 95 female cases (0.05%, 95/200654), with a male/female ratio of 7.9:1 for G6PD deficiency, indicating a significant difference between genders in the prevalence of G6PD deficiency (χ^2^ = 518.581, *p* = 0.000). The median value for G6PD activity was 1.31 U/g Hb for male newborns, and 2.21 U/g Hb for females newborns.

### Sequencing and G6PD/6PGD results

A total of 768 cases were recalled to collect the blood samples for genotype analysis and quantitative G6PD enzymatic assay. The median value for G6PD/6PGD ratio was 0.27 for male, and 0.97 for females. Among the 768 G6PD deficient samples, 694 patients (630 males and 64 females) were detected to have *G6PD* gene variants, while the remaining 74 cases (49 males and 25 females) had no identifiable variant. The number and frequency of different *G6PD* genotypes in the Wuhan cohort are listed in Table 2, with 630 males as hemizygotes, 63 females as heterozygotes, and 1 as homozygote. Meanwhile, 38 kinds of G6PD variants were identified from 694 patients, with 26 variants as single nucleotide substitutions, 1 variant as synonymous variant, 2 variants as deletion, and 11 variants as multiple mutations (double heterozygote/compound heterozygous mutation). The top five common genotypes identified in this study were Kaiping (c.1388 G > A) and Canton (c.1376 G > T), followed by Gaohe (c.95 A > G), Chinese-5 (c.1024C > T) and Viangchan (c.871G > A), accounting for 86.46% (600/694) of all the G6PD deficiency samples. However, the most common variant sites c.383T > C, c.493A > G, c.1381G > A and c.1387C > T in Chinese were not detected in this cohort. In the present study, all the 49 subjects with c.871G > A variant were combined with the c.1311 C > T/1365-13T > C polymorphism, and 11 (7 females and 4 males) of them had only c.1311C > T/1365-13T > C polymorphism.

**TABLE 2:** The number and frequency of different G6PD genotypes in the Wuhan cohort.

Variant name	Genotypes	gDNA nt position	Amino acid substitution	Exon	Hemizygous mutation	Homozyous mutation	Heterozygous mutation	Total	Frequency (%)	enzyme activity (U/g Hb)	G6PD/6PGD ratio
Kaiping	c.1388 G > A	13896	p.Arg463His	12	200	1	13	214	30.79	1.47	0.28
Canton	c.1376 G > T	13884	p.Arg459Leu	12	197	0	17	214	30.79	0.95	0.16
Gaohe	c.95 A > G	95	p.His32Arg	2	70	0	4	74	10.65	1.03	0.26
Chinese-5	c.1024 C > T	13184	p.Leu342Phe	9	48	0	1	49	7.05	2.00	0.50
Viangchan	c.871G > A/c.1311C > T/ IVS-11 93 T > C	13031/ 13714	p.Val291Met/p.Tyr437	9/11	46	0	3	49	7.05	1.48	0.36
Mahidol	c.487 G > A	11658	p.Gly163Ser	6	9	0	1	10	1.44	1.43	0.37
Nankang	c.517 T > C	11688	p.Phe173Leu	6	7	0	0	7	1.01	1.68	0.42
Qing Yuan	c.392 G > T	10892	p.Gly131Val	5	5	0	0	5	0.72	1.95	0.38
	c.1311 C > T/IVS-11 93 T > C	13714	p.Tyr437	11	4	0	7	11	1.58	2.17	0.70
Union	c.1360 C > T	13763	p.Arg454Cys	11	4	0	0	4	0.58	0.43	0.03
Mediterranean	c.563 C > T	11734	p.Ser188Phe	6	4	0	0	4	0.58	0.82	0.01
Shunde	c.592 C > T	11763	p.Arg198Cys	6	4	0	1	5	0.72	0.43	0.67
	c.25 C > T	25	p.Arg9Trp	2	3	0	0	3	0.43	2.17	0.55
	c.152 C > T	10008	p.Thr51Ile	3	3	0	0	3	0.43	2.22	0.45
Aures	c.143 T > C	9999	p.Ile48Thr	3	2	0	0	2	0.29	1.9	0.54
Chinese-1	c.835 A > T	12548	p.Thr279Ser	8	2	0	0	2	0.29	2.00	0.46
Montalbano	c.854 G > A	12567	p.Arg285His	8	2	0	0	2	0.29	1.50	0.37
Fushan	c.1004 C > A	13164	p.Ala335Asp	9	2	0	0	2	0.29	2.38	0.50
Kamogawa	c.169C > T	10120	p.Arg57Trp	4	1	0	0	1	0.14	1.98	0.25
Songklanagarind	c.196T > A	10147	p.Phe66Ile	4	1	0	0	1	0.14	0.98	0.91
Valladolid	c.406C > T	10906	p.Arg136Cys	5	1	0	0	1	0.14	2.44	0.7
Naone	c.497 G > A	11668	p.Arg166His	6	1	0	0	1	0.14	2.48	0.21
Radlowo	c.679 C > T	12027	p.Arg227Trp	7	1	0	0	1	0.14	2.01	0.26
Ierapetra	c.1057 C > T	13356	p.Pro353Ser	10	1	0	0	1	0.14	2.47	0.94
	c.406C > T/c.1311C > T/ IVS-11 93 T > C				1	0	0	1	0.14	1.71	0.47
	c.143T > A/c.1311C > T/ IVS-11 93 T > C				1	0	0	1	0.14	1.08	0.44
	c.1388G > A/871G > A/ c.1311C > T/IVS-11 93 T > C				1	0	0	1	0.14	1.38	0.46
	c.1004 C > A/c.1388 G > A				0	0	1	1	0.14	1.42	0.78
	c.1024 C > T/c.1388 G > A				0	0	1	1	0.14	0.64	0.23
	c.392 G > T/c.1376 G > T				0	0	1	1	0.14	1.52	0.53
	c.1376G > T/ c.1388G > A				0	0	1	1	0.14	0.98	0.21
	c.1376G > T/c.1311C > T/ IVS-11 93 T > C				0	0	1	1	0.14	1.94	0.82
	IVS-5 637/638Tdel/ 1311C > T/IVS-11 93 T > C				0	0	1	1	0.14	2.23	0.80
	IVS-5 637/638Tdel				4	0	10	14	2.01	2.37	0.57
	c.49 C > T	49	p.Arg17Trp	2	1	0	0	1	0.14	1.77	0.46
	c.691 G > A	12039	p.Ala231Thr	7	1	0	0	1	0.14	2.24	0.33
	c.857 A > T	12570	p.Asp286Val	8	1	0	0	1	0.14	0.23	0.01
	c.982 G > A	13142	p.Val328Met	9	2	0	0	2	0.29	2.19	0.44

Seven rare single variants and two rare multiple variants were found in the present cohort, including 3 cases of c.25C > T, 3 cases of c.152C > T, 1 case of c.406C > T, 1 case of c.497G > A, 1 case of c.679C > T, 2 cases of c.854G > A, and 1 case of c.1057C > T. Among them, the new variants c.497G > A, c.679C > T and c.1057C > T had never been reported in China before. The two rare multiple variants included 1 compound heterozygous variant IVS-5 637/638 T del/c.1311C > T/1365-13T > C detected in a female newborn and 1 compound hemizygous variant c.406C > T/c.1311C > T/1365-13T > C detected in a male G6PD deficient sample. Based on the information collected from Human Gene Mutation Database (HGMD), Online Mendelian Inheritance in Man (OMIM), 1000 Genomes(1000G), Exome Aggregation Consortium (ExAC), and ClinVar public databases, four novel *G6PD* variants (c.49C > T, c.691G > A c.857A > T and c.982G > A) were detected in the present cohort, which have never been reported before.

### Results of wilcoxon rank sum test

**FIGURE 1:**
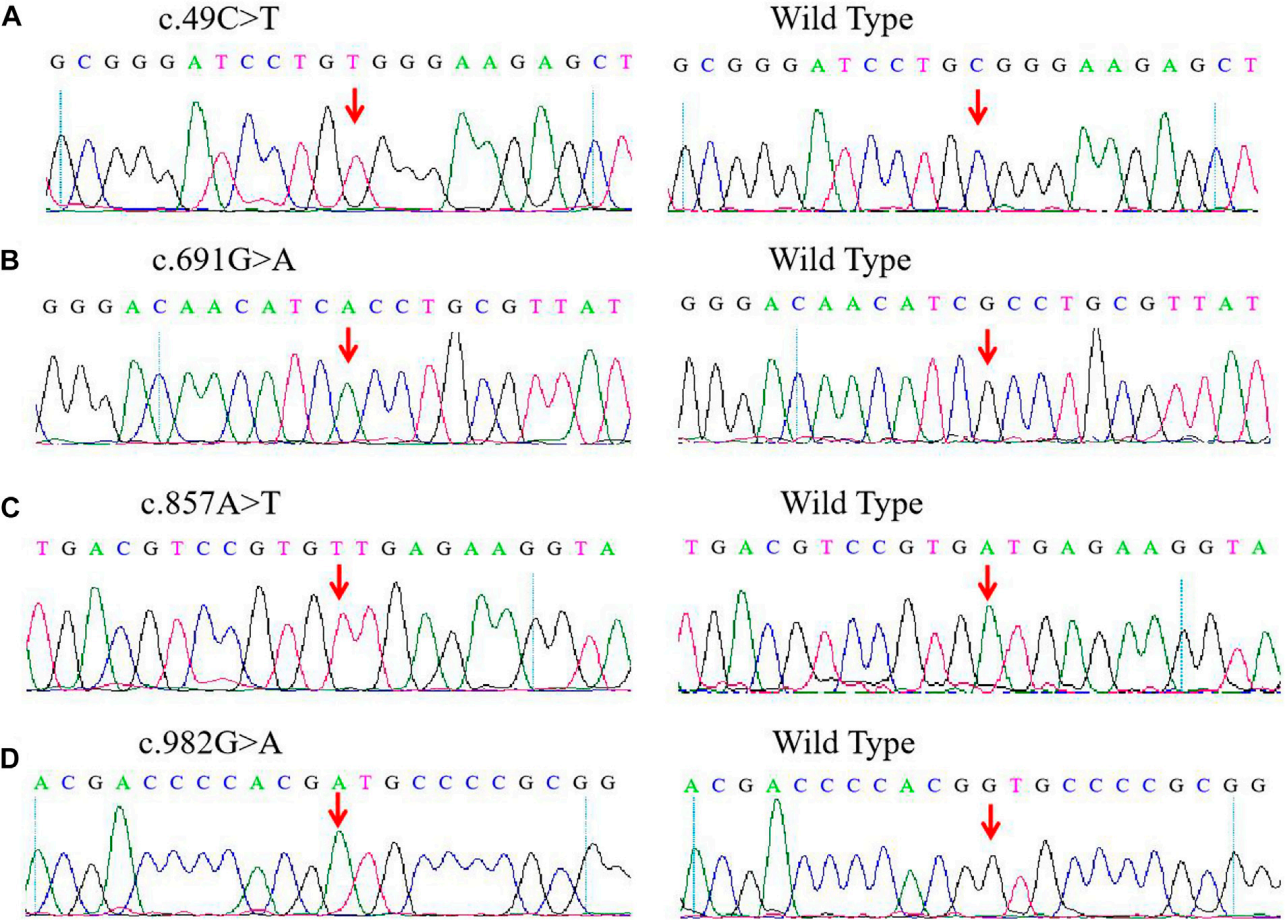
Detection of four novel G6PD variations by Sanger sequencing. **(A)** *c.DNA 49C > T* site in exon 2. **(B)** *c.DNA 691G > A* site in exon 7. **(C)** *c.DNA 857A > T* site in exon 8. **(D)** *c.DNA 982G > A* site in exon 9. The red arrow indicates the location of the mutations.

For the four novel *G6PD* variants (Figure 1), c.49C > T was found in exon 2 and related to the substitution of arginine for tryptophan at position 17 (p.Arg17Trp); c.691G > A was located in exon 7 and associated with the substitution of the alanine231Threonine (p.Ala231Thr) amino acid; c. 857A > T was located in exon 8 and induced the substitution of amino acid aspartic acid for valine at position 286 (p.Asp286Val); c.982G > A was located in exon 9 and related to the replacement of amino acid valine in position 328 by Methionine (p.Val328Met).

**FIGURE 2:**
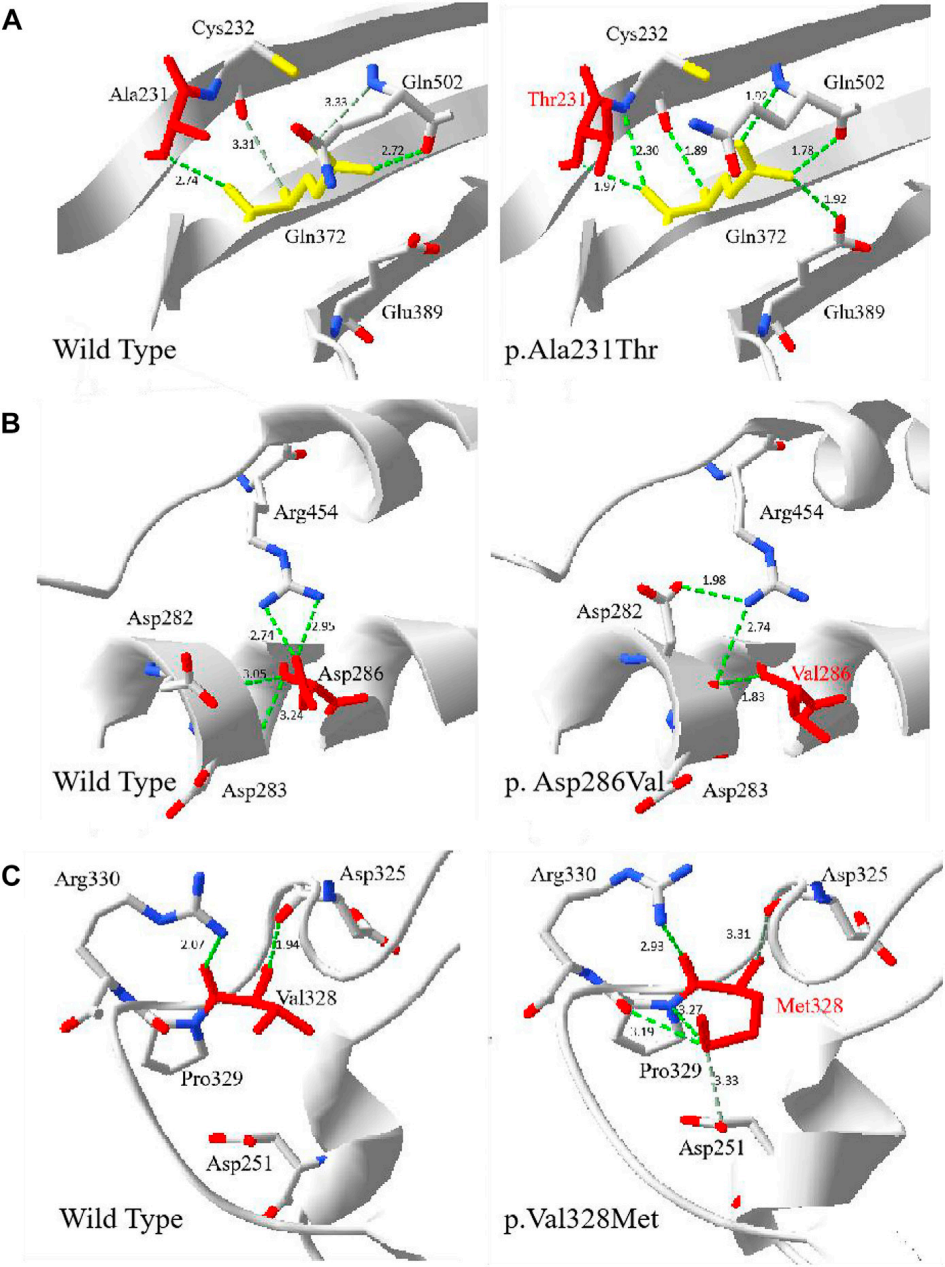
The 3D evaluation of the G6PD variants protein. The crystal structure of the human G6PD was obtained from PDB (PDI id: 2BH9 and 2BHL) . Green dot lines represent hydrogen bonds. Grey dot lines represent weak hydrogen bonds. The atoms of amino acids were colored by type (carbon was grey, oxygen was red, and nitrogen was blue). **(A)** The wild type and p.Ala231Thr G6PD. The residue Thr231 was shown in red. **(B)** The wild type and p. Asp286Val G6PD. The residue Val286 was shown in red. **(C)** The wild type and p.Val328Met. The residue Met 328 was shown in red.

Multiple G6PD amino acid sequence alignment revealed that residues p.Arg17, p.Ala231, p.Asp286 and p.Val328 were highly conserved among mammalian species. The four novel variants (c.49C > T, c.857A > T, c.691G > A and c.982G > A) were predicted to be disease-causing by Mutation Taster, with a score of 0.999, respectively. Based on PolyPhen-2 prediction, p.Arg17Trp was benign, while p.Ala231Thr, p.Asp286Val and p.Val328Met were probably damaging for the G6PD function. Moreover, SIFT indicated that all the four amino acid changes were deleterious. The influences of p.Ala231Thr, p. Asp286Val and p.Val328Met on the three-dimensional (3D) structure were analyzed using the Swiss-Model and Swiss-PdbViewer (Figure 2). A comparison of the model structures of the wild type and the p.Ala231Thr variant indicated that the substitution did not change the hydrogen-bonding between the wide-type Ala 231 and Gln372, but led to the formation of two additional hydrogen-bonds between the Gln372 and its surrounding amino acid residues (Cys232 and Glu389). Analysis of the 3D structure of p.Asp286Val showed that this variant destroyed two hydrogen bonds between the side chains of Asp286 and Arg454 and one hydrogen bond between the side chains of Asp286 and Asp283. Additionally, the p.Val328Met substitution showed that Met328 formed two additional hydrogen bonds with Asp251 and Pro329.

## Discussion

G6PD, a key regulatory enzyme in the pentose phosphate pathway and an active cytosolic enzyme in red blood cells (RBCs), exists in various forms (Luzzatto and Arese 2018). The mutation of *G6PD* gene can lead to the deficiency of activity, weaken the role in regulation of redox state, and cause a clinically common disease-G6PD (Lee et al., 2022). Genetic analysis of G6PD-deficient patients indicated that different ethnic groups varied in their characteristic profiles of G6PD-deficiency variants (Nkhoma et al., 2009). The pattern of G6PD deficiency was geographically consistent with historical prevalence and synchronously varied with the incidence of malaria in China. The frequency of G6PD deficiency and the selective advantage against malaria had a negative correlation with the latitudes, which was consistent with the report that the incidence of G6PD in China was characterized by a gradient distribution from high in South to low in North (Zheng et al., 2020). Wuhan is located at in the mid-latitude region, a middle malaria incidence area, and its G6PD carrying situation may be very rich due to the central location of a transportation hub. In the present study, we evaluated the *G6PD* gene variant distribution in Wuhan District by screening 430,806 neonates born from November 2016 to December 2021, and the G6PD deficiency rate was 0.22% (958/430,806), with an increase in the prevalence of G6PD deficiency in Wuhan birth cohort relative to the prevalence (0.18%) in our previous study (Shen et al., 2019). A possible explanation is that in recent years, the population structure and size have changed greatly, and the increase of urban population from all over the country has induced a more complex genetic background with a rapid floating characteristic, leading to an increase of G6PD deficiency prevalence. However, from the data comparing the disparity in the frequency of G6PD-deficient alleles between male cases (0.37%, 863/230152) and 95 female cases (0.05%, 95/200654) obtained in our study, it’s seemed that quite a few heterozygous female neonates may be misdiagnosis. G6PD deficiency is an X-linked inherited disorder that largely affects hemizygous males and homozygous females, whereas heterozygous females present normal, intermediate or deficient G6PD activity due to random chromosome X inactivation. Routine newborn screening of G6PD deficiency applies one cut-off value (2.5 U/g Hb) could induce misdiagnosis of a considerable proportion of heterozygous female neonates with probable partial G6PD deficiency in clinical practice. It had been reported that increased the cut-off value to 3.8 U/g Hb for G6PD activity could reduced the misdiagnosis rates in heterozygous female that older than 3 months (Peters et al., 2017). Thus a laboratory-specific cut-off values for neonatal screening of G6PD deficiency needs to be established to increase the sensitivity in detecting female heterozygotes.

Mutations in G6PD deficiency were nearly all single nucleotide substitutions, with a few exceptions including small deletions, multiple mutations, and stop codon mutations. In the present study, 38 kinds of *G6PD* variants were identified, including 26 single nucleotide substitutions, 2 deletions, and 11 compound mutations, and among them, there were 7 rare single variants, 2 rare multiple variants, and 4 novel variants. G6PD Canton (c.1376G > T, 30.79%), G6PD Kaiping (c.1388G > A, 30.79%) and G6PD Gaohe (c.95A > G, 10.65%) were the most common *G6PD* variants in the Wuhan region, accounting for 72.23% of total disease alleles, close to the data in the other provinces of China, such as Fujian (72.67%), Guangdong (81.5%), Yunnan (78.6%), Guizhou (79.4%), and Taiwan (78.7%) (Chen et al., 2018). c.1388G > A, c.1376G > T and c.95A > G were the main type of *G6PD* gene variants in the Chinese population, and these three variants were reported to have occurred before the formation of these Chinese ethnic groups (Zheng et al., 2020). After the three variants were the two single variants of Chinese-5 (c.1024C > T, 7.05%), and Viangchan (c.871G > A, 7.05%). Importantly, the five most frequent G6PD gene variants in the Wuhan region accounted for 86.46% of our G6PD-deficient subjects, which agreed with the proportions in the provinces of Sichuan, Chongqing, Guizhou, Hunan, Guangxi, Guangdong, and Hainan (Liu et al., 2020). China has a vast territory, and different regions have their own characteristics (Mason et al., 2007), resulting in variations of *G6PD* gene types and proportions in different geographical locations. For example, Mahidol c.487G > A was quite common in Shandong and Shanxi provinces, but rare in the provinces of Zhejiang, Guangxi, and Guangdong (Liu et al., 2020). In Wuhan neonates, Mahidol c.487G > A accounted for 1.44% (10/694), ranking sixth among the most common sites. Coimbra c.592C > T and Union c.1360C > T were rarely detected in the newborns from the provinces of Sichuan, Congqing, Hunan, Guizhou, and Shanxi, but detected in the newborns from the coastal areas, such as Shandong, Zhejiang, Guangdong, and Guangxi. The frequency of those two variants was 0.72% and 0.58% in Wuhan neonates. Compared with the other provinces of China, Wuhan area showed several regional features in *G6PD* gene pathogenic variants. Specifically, some rare variants were found in the Wuhan cohort, such as c.25C > T, c.152C > T, Valladolid c.406C > T, Naone c.497G > A, Radlowo c.679C > T, Montalbano c.854G > A, FIerapetra c.1057C > T, c.406C > T/c.1311C > T/1365-13T > C, and IVS-5 637/638 T del, with c.25C > T, c.152C > T, c.406C > T, c.854G > A, c.406C > T/c.1311C > T/1365-13T > C, and IVS-5 637/638 T del being sporadically reported in the Chinese population previously (Shen et al., 2017; Chen et al., 2018; Liu et al., 2020). Note that the new variant c.497G > A was first discovered in Southwestern Pacific, resulting in the replacement of the Arg at 166 site by His, c.679C > T was first discovered in Poland, resulting in the replacement of the Arg at 227 site by Trp, c.1057C > T was first discovered in Greece, resulting in the replacement of the Pro at 353 site by Ser, which were first identified in the Chinese population in the present study (Minucci et al., 2012; Luzzatto et al., 2020). Meanwhile, four common Chinese variants (c.383T > C, c.493A > G, c.1381G > A, and c.1387C > T) were not detected in the present study. These findings reflected the variety of variants in the Wuhan region as well as their own regional characteristics.

The 4 novel *G6PD* variants [c.49C > T (p.Arg17Trp), c.857A > T (p.Asp286Val), c.691G > A (p. Ala231Thr), and c.982G > A (p. Val328Met)] were first discovered in Wuhan and they have never been reported in other variant databases. For the novel variant p.Arg17Trp, the positively charged-polar amino acid Arg was substituted by the aromatic amino acid Trp, which was located in the N-terminal region. As previously reported (Kotaka et al., 2005), the 25 N-terminal residues of human G6PD preceding the dinucleotide-binding fold were removed, but little is known about the effect of the 25 removed residues on G6PD enzyme function. Some reports mentioned that a change in residues located at the 25 N-terminus may result in G6PD enzyme deficiency (Minucci et al., 2012; Luzzatto et al., 2020). One heterozygous female was found to be deficient who showed low normal G6PD activity (1.77 U/g Hb), G6PD/6PGD ratio was 0.46. According to WHO classification, the p.Arg17Trp was classified as class Ⅲ. Substitution of nucleotide 691 was previously reported in Shanghai, and c. 691 G > C (p.Ala231Pro) variant was shown to be associated with highly severe reduction of G6PD activity (Wang et al., 2020). Regarding our patient, the p.Ala231Thr substitution can be speculated to be more deleterious, since Ala is a non-polar amino acid replacing the polar amino acid Thr and is predicted to introduce additional polar contacts with the nearby amino acid, thereby over-stabilizing the protein structure. The location of the novel variant p. Ala231Thr was neither part of the substrate or structural NADP^+^ binding sites nor localized close to the dimer/tetramer interface, however it lead to the change of hydrogen-bonds between the Gln372 and its surrounding amino acid residues (Cys232, Glu389 and Gln502). The amino acids Gln372 at the dimer interface, Glu389 close to the dimer interface and close to the structural NADP^+^ molecule. The p.Gln502, which is a part of the NADP^+^-binding pocket. The G6PD activity of one heterozygous female with the variant p. Ala231Thr was 2.24 U/g Hb, G6PD/6PGD ratio was 0.33. Therefore, according to WHO classification, the p. Ala231Thr variant was classified as class Ⅲ. For the novel variant p.Asp286Val, the negatively charged amino acid (Asp) replaced the non-polar amino acid (val), this was predicted to cause loss of polar contacts with the nearby amino acids, which was predicted to cause the loss of two hydrogen bonds between the residue at position 286 and the nearby amino acids, Asp283 and Arg454, indicating the loss of protein stability. G6PD activity of one heterozygous female with the variant p.Asp286Val was 0.23 U/g Hb, G6PD/6PGD ratio was 0.01, according to WHO classification, the p.Asp286Val variant was classified as class Ⅱ. For the p.Val328Met variant, both Val and Met were the non-polar amino acids. However, two new hydrogen bonds were formed between Met328 and the residues of Pro329 and Asp251. Two heterozygous females carrying this variant were found to be deficient, while the mean of G6PD activity was 2.19 U/g Hb, G6PD/6PGD ratio was 0.44, according to WHO classification, the p. Val328Met variant was classified as class Ⅲ. It had been reported that mutations causing severe deficiency are concentrated close to the structural NADP^+^ and the dimer interface, indicating that the integrity of these regions is important for enzyme stability and therefore for its *in vivo* activity. In the meanwhile, the protein stability is a key factor for the functionality of protein (Au et al., 2000; Rani et al., 2022). 3D structure analysis of p.Ala231Thr, p.Asp286Val, and p.Val328Met also showed that the main influences were the changes of the hydrogen bonding between the side chains of amino acid variants and the surrounding amino acids, leading to the destabilizing or overstabilizing of the protein structure, which indicated that the amino acid replacement could generate a conformational change on protein stability and enzymatic activity.

In conclusion, this is the first report of a systematic survey of G6PD deficiency and associated variants in the neonates in the Wuhan area. In the present study, we determined the frequency of common *G6PD* variants and revealed seven rare variants and four novel *G6PD* variants in the Wuhan area. These results indicated a rich diversity of *G6PD* genetic mutations with regional characteristics in the Wuhan cohort. This study not only provides the basic knowledge regarding the genotype and distribution characteristics of G6PD deficiency, but also reference data for diagnosis and research of G6PD deficiency in the Wuhan region. In the future study, it is necessary and important to establish the cut-off values of G6PD in Wuhan for neonates G6PD screening to increase the sensitivity in detecting female heterozygotes.

## Data Availability

The datasets presented in this study can be found in online repositories. The names of the repository/repositories and accession number(s) can be found below: ON843698-ON843701.
